# Neo-adjuvant Capecitabine Chemotherapy in Women with Newly Diagnosed Locally Advanced Breast Cancer in a Resource-poor Setting (Nigeria): Efficacy and Safety in a Phase II Feasibility Study

**DOI:** 10.1111/tbj.12149

**Published:** 2013-07-19

**Authors:** Olukayode A Arowolo, Uchenna O Njiaju, Temidayo O Ogundiran, Oyewale Abidoye, Olukayode O Lawal, Millicent Obajimi, Adebayo V Adetiloye, Hae K Im, Akinbolaji A Akinkuolie, Abideen Oluwasola, Kayode Adelusola, Adesunkanmi A Kayode, Augustine E Agbakwuru, Helen Oduntan, Chinedum P Babalola, Gini Fleming, Olusola C Olopade, Adeyinka Gladys Falusi, Muheez A Durosinmi, Olufunmilayo I Olopade

**Affiliations:** * Department of Surgery, College of Health Sciences, Obafemi Awolowo UniversityIle-Ife, Osun, Nigeria; † Section of Hematology/Oncology, Department of Medicine, The University of Chicago Medical CenterChicago, Illinois; ‡ Department of Surgery, College of Medicine, University of IbadanIbadan, Oyo, Nigeria; § Department of Radiology, College of Medicine, University of IbadanIbadan, Oyo, Nigeria; ¶ Department of Radiology, College of Health Sciences, Obafemi Awolowo UniversityIle-Ife, Osun, Nigeria; ** Department of Health Studies, The University of ChicagoChicago, Illinois; †† Department of Pathology, College of Medicine, University of IbadanIbadan, Oyo, Nigeria; ‡‡ Department of Haematology and Immunology, College of Health Sciences, Obafemi Awolowo UniversityIle-Ife, Osun, Nigeria; §§ Pharmacy Department, University College HospitalIbadan, Oyo, Nigeria; ¶¶ Department of Pharmaceutical Chemistry, Faculty of Pharmacy, University of IbadanIbadan, Oyo, Nigeria; *** Genetics and Bioethics Unit/Breast Cancer Laboratory, Institute for Advanced Medical Research and Training, College of Medicine, University of IbadanIbadan, Oyo, Nigeria; ‡‡‡ Center for Global Health, The University of ChicagoChicago, Illinois

**Keywords:** capecitabine, clinical response, locally advanced breast cancer, Nigeria, resource-poor country

## Abstract

The majority of clinical trials of neo-adjuvant therapy for breast cancer have been conducted in resource-rich countries. We chose Nigeria, a resource-poor country, as the major site for a phase II feasibility open-label multicenter clinical trial designed to evaluate the efficacy, safety, and tolerability of neo-adjuvant capecitabine in locally advanced breast cancer (LABC). Planned treatment consisted of 24 weeks of capecitabine at a dose of 1,000 mg/m^2^ twice daily (2,000 mg/m^2^ total per day). The primary endpoints were overall, partial, complete clinical response rate (OCR, PCR, CCR) and complete pathologic response (cPR). A total of 16 patients were recruited from August 2007 to April 2010. The study was terminated early as a result of slow accrual. After the first three cycles of therapy, PCR were seen in five of 16 patients (31%; 95% CI 11–59%). Of the remaining 11 patients, eight had no response (NR) or stable disease (SD), and three had progressive disease (PD). Seven patients proceeded with further therapy of which had SD. OCR at the end of eight cycles was 44% (95% CI 20–70%). Clinical response and radiologic response by ultrasonomammography were highly concordant (spearman correlation 0.70). The most common adverse effect was Grade 1 hand–foot syndrome, which was seen in 75% of patients. Despite several limitations, we successfully carried out this phase II feasibility study of neo-adjuvant capecitabine for LABC in Nigeria. Capecitabine monotherapy showed good overall response rates with minimal toxicity and further studies are warranted.

Breast cancer is a major global health problem. It is not only the most common cancer, but has the highest mortality rate among women in African countries including Nigeria 1–3. Among women born and raised in the USA, black women have a lower risk of breast cancer than white women, but the survival rate of the former group is poorer probably due to advanced stage at diagnosis and histologically more aggressive disease 4,5. African-American patients have a greater incidence of breast cancer between 30 and 44 years (which is less likely to be screen-detected) and their tumors are more likely to be poorly differentiated and estrogen receptor (ER) negative, with high nuclear atypia and higher S-phase fraction 4.

In West Africa, the founder population of most African Americans, breast cancer is considered to be a rare, virulent disease of young women 6,7. Breast cancer in these young women is almost uniformly fatal, in part, due to ignorance about the disease and poor access to modern medical care. In Nigerian women, the mean age at presentation is 43 years, 74% of affected individuals are premenopausal, and 12% are under the age of 30 1,7,8.

Guidelines for the treatment of locally advanced breast cancer (LABC), with particular reference to resource-poor countries, have been published 9. The Breast Health Global Initiative (BHGI) summit in 2007 focused on the management and implementation of primary systemic therapy in the form of neo-adjuvant chemotherapy in LABC and two important points were highlighted 10. Firstly, all trials demonstrating the efficacy of neo-adjuvant chemotherapy in breast cancer have been conducted in developed countries. Secondly, there is a paucity of literature on the impact of neo-adjuvant chemotherapy for LABC in developing countries such as Nigeria and although scientific advances drive management guidelines, the implementation is limited by local resources and expertise 10–12.

Although newer agents are being studied in resource-rich nations 13,14, neo-adjuvant chemotherapy for LABC remains predominantly anthracycline-based, particularly in resource-poor nations 12. In spite of high response rates to anthracycline-based regimes, only a small fraction of patients achieve a complete pathologic response (cPR); more than 60% of patients with LABC continue to die of metastatic breast cancer; and disease-free survival (DFS) rates remain modest at only about 30% at 5 years 2,9. Clearly, further refinement of treatment strategies and introduction of more effective and less toxic chemotherapeutic agents are warranted. It is conceivable that a less toxic regimen given for longer duration might lead to more cPRs and improved quality of life as has been demonstrated in patients with hormone receptor-positive tumors treated with prolonged duration of hormonal therapy. The identification of effective agents that can be used alone or in combination with other agents is uniformly accepted as an appropriate drug development strategy in breast cancer. Capecitabine is preferentially taken up by cancer cells and represents an important new class of drugs that deserves testing in chemotherapy naïve patients with LABC 15.

The rationale of this study was to test the clinical efficacy of capecitabine given as neo-adjuvant therapy in patients with LABC. The hypothesis to be tested was that single-agent capecitabine chemotherapy given at 1,000 mg/m^2^ twice daily (2,000 mg/m^2^ per day) will result in acceptable overall response rates (ORR) and minimal toxicity in chemotherapy naive LABC. In addition, the study was designed to demonstrate feasibility of a phase II trial involving an oral chemotherapeutic agent such as capecitabine in a resource-poor nation like Nigeria.

## Patients and Methods

### Patient Eligibility

The study was opened in Chicago and in two sites in Nigeria, under the auspices of the University of Chicago Center for Global Health (CGH). Patients with newly diagnosed LABC were seen at the three institutions involved in the study, namely: The University of Chicago Comprehensive Cancer Center, Chicago; Obafemi Awolowo University Teaching Hospitals Complex (OAUTHC), Ile-Ife, Nigeria; and the University College Hospital (UCH), Ibadan, Nigeria. Patients were enrolled after meeting eligibility criteria. Eligible patients were women with newly diagnosed and histologically confirmed, locally advanced adenocarcinoma of the breast (T3-4b, N0-3, M0 disease). Patients with asymptomatic bone metastases and patients with large T2 tumors, whose surgeons believed that results with breast-conserving surgery would be improved by neo-adjuvant therapy, were also eligible. Patients were 18 years or older, had a negative serum or urine pregnancy test within 7 days prior to starting therapy if considered to be of child-bearing potential, and had an Eastern Cooperative Oncology Group (ECOG) performance status of 0–1. Other eligibility criteria included lack of prior therapy for the index malignancy and adequate organ function as shown by an absolute neutrophil count ≥1,500/μL, platelet count ≥100,000/μL, creatinine ≤1.5 mg/dL, calculated creatinine clearance ≥50 mL/min, total bilirubin ≤1.4 mmol/L, aspartate aminotransferase/alanine aminotransferase ≤1.5× upper limit of normal, and alkaline phosphatase ≤2.5× upper limit of normal.

All patients completed a written consent, with those recruited in Nigeria having their data transmitted to a data-monitoring site at the University of Chicago. The study protocol was approved by the Institutional Review Board at each participating institution.

### Study Design and Patients Selection

The study was a multicenter, single-arm, phase II feasibility trial of neo-adjuvant chemotherapy with capecitabine in women with newly diagnosed LABC. Initial assessment of patients included a medical history, physical examination, complete blood count with differential, a metabolic panel, chest x-ray, electrocardiogram, bilateral breast mammogram, pregnancy test, and pathologic review of tissue. Immunohistochemistry was used to assess the following tumor markers: ER, progesterone receptor (PR), and the HER2-neu receptor. ER and PR status were considered positive if >10% of any sample stained positive. Tumor grade was also assessed. Additional studies were performed as clinically indicated. Women without menstrual bleeding over the preceding 12 months were considered to be postmenopausal.

### Treatment Regimen

Enrolled patients were treated for a maximum of eight cycles with capecitabine at a dose of 1,000 mg/m^2^ twice daily (2,000 mg/m^2^ total per day) for 2 weeks followed by a 1-week pause. Each cycle lasted for 21 days. Final evaluation was performed by week 26 and no later than week 28. Imaging studies were performed no more than 28 days before initiation of therapy, and all laboratory work was completed no more than 16 days before registration. As the study was conducted in resource-poor settings, ultrasonomammography was a cost-effective method for assessing tumor response and was used in the study. Definitive surgery with modified radical mastectomy (simple mastectomy and axillary lymph node dissection (ALND) was completed between weeks 28 and 30, but not later than week 32). Following completion of chemotherapy, patients with ER+ or PR+ tumors received tamoxifen at a dose of 20 mg orally once daily, or an aromatase inhibitor if postmenopausal. Anti-estrogen therapy was started within 12 weeks of completion of chemotherapy and continued for 5 years. Patients who received radiation therapy to the chest wall and/or nodal areas started treatment 6–8 weeks after completion of definitive surgery.

### Patient Assessment and Outcome Measurement

History with toxicity assessment using Common Toxicity Criteria version 2.0 16, and a physical examination with tumor measurement were done at the start of each cycle. A mammogram and breast ultrasound were done prior to initiation of therapy and after the 3rd and 8th cycles, or earlier if clinically indicated. Partial response was defined as reduction by at least 50% of the sum of the products of the longest perpendicular diameters of all measurable lesions. Complete clinical response (CCR) was defined as complete disappearance of all measurable malignant disease, and no new malignant lesion, disease-related symptoms, or evidence of evaluable disease. No response (NR) was defined as reduction less than 25% of the sum of the products of the longest perpendicular diameters of all measurable lesions as determined by two observers at least 3 weeks apart, whereas stable disease (SD) was defined as decrease between 25 and 50% the sum of the products of the longest perpendicular diameters of all measurable lesions. The same percentage changes in size were used to determine response on ultrasonomammography. pCR was defined as the absence of invasive breast cancer in the breast (mastectomy or lumpectomy) specimen at the time of definitive surgery.

### Statistical Analysis

The trial was originally designed in two stages that would allow early stopping for lack of efficacy. In the first trial stage, 21 patients would be accrued, treated, and assessed. If fewer than eight patients had a response, then the trial would be discontinued for lack of efficacy. If there were eight or more responses observed, then an additional 27 patients would be enrolled. Thus, the maximum sample size was 48 patients. If there had been 20 or more responses among the 48 patients, then the trial results would have been considered supportive of a response proportion of 50%. Under this trial design, power to detect a response difference of the magnitude specified is 0.85, and the probability of falsely concluding in favor of the alternative (e.g., the alpha level) is 0.05. The probability of early stopping if indeed the response rate was 30% is 0.72. As the trial was terminated due to slow accrual, we report frequency of responses with 95% exact confidence intervals assuming binomial distribution of events.

## Results

Between August 2007 and April 2010, a total of 18 patients were enrolled in the study, including one patient treated at the University of Chicago. Two patients were withdrawn before commencement of the study as a result of inconclusive histopathology results and voluntary default, respectively. Patients’ demographic and tumor characteristics are summarized in Tables1 and 2.

**Table 1:** Baseline Patient Characteristics

Characteristic	*N*	Percentage (%)
Age		
Mean	50.1	
SD	11	
Range	32–70	
Race		
Black African	15	93.7
Black american	1	6.3
Weight		
Mean	69.2	
SD	14.4	
Median	70.5	
Range	47–94	
Performance status		
0	14	87.5
1	2	12.5
Measurable disease		
Yes	16	100
No	0	0
Patients occupation		
Civil servant	3	18.6
House wife	1	6.3
Petty trader	10	63.5
Teacher	2	12.5
Primary cancer site		
Left breast	11	68.7
Right breast	5	31.3
Clinical stage of cancer		
T3	12	75
T4c	1	6.3
T4a	2	12.5
T2	1	6.3
N0	2	12.5
N1	9	56
N2a	4	25
N3a	1	6.3

**Table 2:** Baseline Tumor Characteristics

Characteristic	*N*	Percentage
Tumor diameter		
Mean	8.9	
SD	2.4	
Median	9.0	
Range	4–15	
Histology		
Invasive ductal carcinoma	14	87
Comedocarcinoma	1	6.3
Mucinous adenocarcinoma	1	6.3
Mitotic index grade		
Grade 1	2	12.5
Grade 2	8	50
Grade 3	6	37.5
Hormone receptor status		
ER+/PR+	1	6.3
ER+/PR−	2	12.5
ER−/PR+	1	6.3
ER−/PR−	12	75
HER2+	0	0
HER2−	16	100

**Figure 1:**
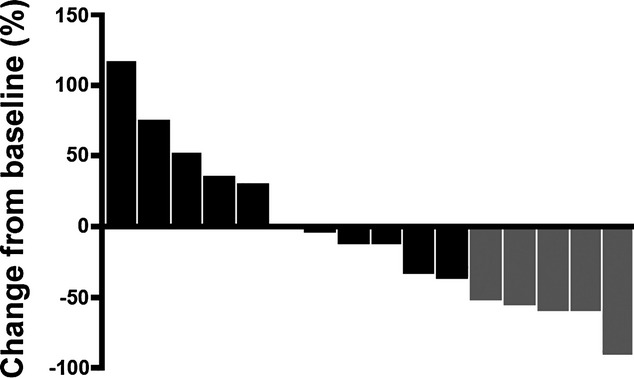
Waterfall plot illustrating patient response to single-agent neo-adjuvant capecitabine. Bars represent the sum of the products of the longest perpendicular diameter of all measurable lesions. Gold bars represent patients with confirmed partial responses, whereas black bars represent patients with stable disease, no response, or progressive disease.

All 16 patients completed the first three cycles of capecitabine chemotherapy, at the end of which five patients (31%) had a partial clinical response (95% CI 11–59). Seven patients continued in the trial and were scheduled to receive a total of eight cycles of capecitabine; however, clinical evaluation after four cycles revealed progressive disease in one patient. One patient was later removed for protocol violation and six patients ultimately completed the planned eight cycles of therapy. Of the six patients who completed eight cycles, two patients had a CCR and four patients had a partial clinical response. Radiological response was also assessed using breast ultrasonomammography. By ultrasound, four patients had partial response of 25% (95% CI 7–52) among 16 patients after three cycles. The median clinical and sonographic tumor size decreased from 63.5 cm^2^ (range 16–180 cm^2^) and 9.2 cm^2^ (range 1.1–103.5 cm^2^) at the beginning of therapy to 9.2 cm^2^ (range 0–16 cm^2^) and 1.7 cm^2^ (range 0.6–6.8 cm^2^) at the end of eight cycles, respectively. Median reduction in bi-dimensional size measured by clinical examination was 34% (100% maximum reduction and 42% maximum increase). Median reduction in bi-dimensional size measured with ultrasound was 34% (92% maximum reduction and 27% maximum increase). Details are shown in Table3 and Fig.1. All six patients had modified radical mastectomies at the completion of the 8th cycle of chemotherapy and no cPR was observed.

**Table 3:** Clinical and Radiological Response at the End of the 3rd and 8th Cycles

	Clinical	Response	USS	Response
	*N* = 16		*N* = 16	
3rd cycle	Number	Percentage		
Complete response	0	0	0	0
Partial response	5	31.3	4	25.0
No response	5	31.3	4	25.0
Stable disease	3	18.8	3	18.8
Progressive disease	3	18.8	5	31.2
Overall response (CR + PR)	5	31.3	4	25.0
Mean tumor area (cm^2^)	50.7		17.3	
Median tumor size (cm^2^)	48.8		8.9	
Range	6 – 143		1.1 – 103.5	
SD	40.8		26.1	
	N = 6		N = 6	
8th cycle				
Complete response	2	13	0	0
Partial response	5	31	5	31.3
No response	0	0	0	0
Stable disease	0	0	0	0
Progressive disease	0	0	0	0
Overall response (CR + PR)	7	43.8	5	31.3
Mean tumor area (cm^2^)	7.7		2.3	
Median tumor area (cm^2^)	9.2		1.7	
Range	0–16		0.6–6.8	
SD	6.7		2.34	

Neo-adjuvant capecitabine at a dose of 1,000 mg/m^2^ was found to be safe and well tolerated in this cohort of patients. None of the patients had grade 2 or 3 toxicities requiring either dosage reduction or cessation of therapy. The most common adverse effect was hand and foot syndrome, which was observed in 75% of patients. Other adverse events recorded during this study included diarrhea, skin rash, fatigue, dizziness, anorexia, and nausea, most of which occurred in less than 10% of patients (Table4). Commonly reported capecitabine-associated toxicities like neutropenia, alopecia, thrombocytopenia, and vomiting were not observed.

**Table 4:** Toxicities

Adverse event	No of patients (*N* = 16)	%
Diarrhea	3	18.8
Skin rash	1	6.3
Hand and foot syndrome	12	75
Fatigue	3	18.8
Dizziness	1	6.3
Anorexia	1	6.3
Nausea	1	6.3
Sore throat	1	6.3
Vomiting	0	0
Alopecia	0	0
Neutropenia	0	0
Thrombocytopenia	0	0

Table5 details outcomes following surgery on the six patients who had surgery after completing eight cycles of capecitabine and two additional patients who progressed after three cycles, but went on to receive additional chemotherapy and surgery. At last follow-up, five of six patients who completed eight cycles of capecitabine are alive and one was lost to follow-up; one of the two patients with progressive disease died of hepatic metastasis 12 months after surgery, whereas the other remained alive and well.

**Table 5:** Surgery and Outcome of Surgery

Patient ID	Age (years)	No of cycles	Response	Surgery done	Length of follow-up (months)	Local recurrence	Systemic recurrence	Present status
011	52	8	PR	MRM	39	Nil	Nil	Alive and Well
008	52	3	PD	MRM	48	Nil	Nil	Alive and well
003	59	8	CR	MRM	7	Nil	Nil	Defaulted from follow-up
018	64	8	PR	MRM	18	Nil	Nil	Alive and well
009	40	8	PR	MRM	21	Nil	Nil	Alive and well
002	48	3	PD	MRM	12	Nil	Hepatic	Died 12 months post op
016	57	8	PR	MRM	36	Nil	Nil	Alive and well
006	47	8	CR	MRM	24	Nil	Nil	Alive and well

PR, partial response; PD, progressive disease; CR, complete response; MRM, modified radical mastectomy.

Figure1 is Waterfall plot illustrating patient response to single-agent neo-adjuvant capecitabine.

## Discussion

To our knowledge, this is the first report of a phase II feasibility study of oral chemotherapeutic agent in the treatment of LABC in Nigeria. The study presented challenges that will need to be addressed and overcome, so as to facilitate more of these clinical trials in the future as such studies will contribute toward improving cancer care in resource-poor nations. To ensure a reasonable sample size for an adequately powered study, we planned to accrue 48 patients over a 2-year period at a rate of two patients per month. The goal seemed reasonable, given the prevalence and increasing incidence of breast cancer in Nigeria, the most populous country in sub-Saharan Africa 1,3. Instead, only 18 patients were accrued and of those, only 16 patients completed the first three cycles of capecitabine chemotherapy. We observed that a large majority of patients presented with advanced disease and were not eligible. Most ineligible patients had features such as exuberant peau d'orange, extensive fungating exophytic breast masses, large tumor size with edematous upper limb, and erythematous skin involvement. Sadly, such patients still make up about 70–80% of breast cancer patients in Nigeria, a pattern which has not changed from the previous decade 1,3. Another prominent factor responsible for our low accrual rate were the health care worker strikes in Nigeria, which disrupted our recruiting and treatment efforts for six of the 32 months of our study.

Capecitabine monotherapy has been extensively studied and found to be effective and safe in the treatment of metastatic breast cancer 17–19. Whether used alone in a monotherapy setting, or used in combination with other chemotherapeutic agents, numerous studies have shown efficacy in metastatic breast cancer with overall clinical benefit rates of 30–70%, ORRs of 15–40%, median time to progression of greater than 3 months, and overall survival in excess of 12 months 20–22. Despite the good response rate seen in the metastatic setting, single-agent efficacy has not been reported in the neo-adjuvant setting for LABC.

Even with low accrual and consequent detrimental effects on study power, we detected certain patterns and trends. The best ORR in this study was 44% (95% CI 20–70%). This study showed that single-agent capecitabine has comparable activity and efficacy with that of single-agent docetaxel in the treatment of patients with LABC. In single-arm phase II clinical trials involving the use of single-agent docetaxel at 100 mg/m^2^, ORRs ranged from 54 to 69%. When used as second-line single-agent therapy, ORRs of 23–65% were observed 23–25. These response rates are similar to the response rates observed in capecitabine monotherapy in this study.

None of our study patients attained a pCR and this may be related to the definition of pCR used. In our study, pCR was defined as no microscopic evidence of residual viable tumor cells, invasive or non-invasive, in all resected specimens of the breast. However, NSABP B-27 and the Aberdeen Trial adopted a loose definition of pCR, which includes the presence of ductal carcinoma in situ (DCIS) in the breast, regardless of the axillary node status 24,25. The ORR of 44% after eight cycles of therapy in this study is comparable with the response rates for metastatic breast cancer treated with capecitabine 20,26. However, the aforementioned rates are lower than ORRs of 40–70% seen with the use of capecitabine in combination with other agents such as docetaxel and bevacizumab 27,28. It is well recognized that combination chemotherapy often results in higher response rates, but with greater toxicity, and higher response rates do not always translate to improved outcomes.

Ultrasonomammography of the breast was used to determine the size of the breast tumor at diagnosis and also to monitor response of the tumor to chemotherapy. There was modest concordance between clinical response and radiological response by breast ultrasound (spearman correlation of 0.70). This high correlation between clinical and ultrasound response rate has also been observed in other studies 29,30. Of note, although CCRs were recorded in two patients with no clinically measurable disease at the end of eight cycles, the breast ultrasound of these two patients still showed residual tumor. This is not surprising as predicting residual tumor size after neo-adjuvant chemotherapy can be challenging 31,32 and physical examination of breast tumors measuring less than 2 cm is often difficult depending on the density of the breast 33. Furthermore, physical examination may be inaccurate when the tumor is irregular or has poorly defined margins or when neo-adjuvant chemotherapy results in residual fibrosis and/or necrosis 34,35.

Capecitabine treatment was well tolerated in this study. Less that 10% of the patients had adverse events except for mild hand–foot syndrome, which was seen in over 70% of the patients studied. The safety profile found in this study was consistent with that seen in previous studies of capecitabine monotherapy in breast cancer 20,21,26,36,37 and colorectal cancer 38,39. The majority of treatment-related adverse events were mild or moderate in intensity and the most frequent adverse event was hand–foot syndrome. This cutaneous condition is characteristic of chronic cytostatic administration, and has been described with other agents such as protracted 5-FU, 96-h vinorelbine, or liposomal doxorubicin. In our patients, hand–foot syndrome was rapidly reversible, such that no patients required treatment interruption. There were no cases of grade 3/4 toxicity in this study and in particular, alopecia and myelosuppression were not observed. The convenient oral administration of capecitabine in combination with its acceptable efficacy and manageable side effect profile makes it an attractive agent for use in an outpatient setting. Oral capecitabine avoids the risk of complications associated with intravenous drug administration and allows patients to control their own therapy and achieve a degree of independence. Based on side effects reported by the participants, it is possible that a higher capecitabine dose could have been tolerated without significant worsening of toxicities. Importantly, for responding patients who went on to surgery, there appears to have been some clinical benefit in improving their outcomes that deserves further evaluation in future studies.

## Conclusions

Neo-adjuvant single-agent capecitabine chemotherapy for LABC in a predominantly Nigerian population is effective and safe with expected toxicities, but no myelosuppression. Clinical trials in low-resource countries are challenging, but these obstacles can be overcome through continued building of resources that offer and support alternative treatments while educating local investigators and participants on the benefits of clinical trials. Increased survival rates from clinical investigation and increased clinical trial participation may translate into improved quality of care for patients with malignant diseases in resource-poor nations. Our study demonstrates that the conduct of clinical trials in a resource-poor nation like Nigeria is feasible, and should be encouraged.

## References

[1] Adebamowo CA, Ajayi OO (2000). Breast cancer in Nigeria. West Afr J Med.

[2] Okobia MN, Osime U (2001). Clinicopathological study of carcinoma of the breast in Benin City. Afr J Reprod Health.

[3] Adesunkanmi ARK, Lawal OO, Adelusola KA, Durosimi MA (2006). The severity, outcome and challenges of breast cancer in Nigeria. Breast.

[4] Layman RM, Thomas DG, Griffith KA (2007). Neoadjuvant docetaxel and capecitabine and the use of thymidine phosphorylase as a predictive biomarker in breast cancer. Clin Cancer Res.

[5] Morris JA, Kelly JF (1982). Multiple bilateral breast adenomata in identical adolescent Negro twins. Histopathology.

[6] Anyanwu SN (2000). Survival following treatment of primary breast cancer in eastern Nigeria. East Afr Med J.

[7] Chiedozi LC (1984). Breast carcinoma in young Nigerian women. Trop Geogr Med.

[8] Chiedozi LC (1987). Rapidly progressing breast cancer in Nigeria. Eur J Surg Oncol.

[9] El Saghir NS, Eniu A, Carlson RW (2008). Locally advanced breast cancer: treatment guideline implementation with particular attention to low- and middle-income countries. Cancer.

[10] Eniu A, Carlson RW, Aziz Z (2006). Breast cancer in limited-resource countries: treatment and allocation of resources. Breast J.

[11] Rastogi P, Anderson SJ, Bear HD (2008). Preoperative chemotherapy: updates of National Surgical Adjuvant Breast and Bowel Project Protocols B-18 and B-27. J Clin Oncol.

[12] Arowolo OA, Akinkuolie AA, Lawal OO, Alatise OI, Salako AA, Adisa AO (2000). The impact of neoadjuvant chemotherapy on patients with locally advanced breast cancer in a Nigerian semiurban teaching hospital: a single-center descriptive study. World J Surg.

[13] Valero VV, Buzdar AU, Hortobagyi GN (1996). Locally advanced breast cancer. Oncologist.

[14] Valero V, Buzdar AU, McNeese M, Singletary E, Hortobaygi GN (2002). Primary chemotherapy in the treatment of breast cancer: the University of Texas M. D. Anderson Cancer Center experience. Clin Breast Cancer.

[15] Stearns V, Singh B, Tsangaris T (2003). A prospective randomized pilot study to evaluate predictors of response in serial core biopsies to single agent neoadjuvant doxorubicin or paclitaxel for patients with locally advanced breast cancer. Clin Cancer Res.

[16] National Cancer Institute (1999). Common Toxicity Criteria Manual version 2.0..

[17] William BE (2006). Capecitabine monotherapy: safe and effective treatment for metastatic breast cancer. Oncologist.

[18] Lee SH, Lee J, Park J (2004). Capecitabine monotherapy in patients with anthracycline- and taxane-pretreated metastatic breast cancer. Med Oncologist.

[19] Pierga JY, Fumoleau P, Brewer Y (2004). Efficacy and safety of single agent capecitabine in pretreated metastatic breast cancer patients from the French compassionate use program. Breast Cancer Res Treat.

[20] O'Shaughnessy JA, Blum JL (2006). Capecitabine/Taxane combination therapy: evolving clinical utility in breast cancer. Clin Breast Cancer.

[21] Reichardt P, Von Minckwitz G, Thuss-Patience PC (2003). Multicenter phase II study of oral capecitabine (Xeloda) in patients with metastatic breast cancer relapsing after treatment with a taxane-containing therapy. Ann Oncol.

[22] Saeki T, Takashima S (2004). Capecitabine plus docetaxel combination chemotherapy for metastatic breast cancer. Breast Cancer.

[23] Figgitt DP, Wiseman LR (2000). Docetaxel: an update of its use in advanced breast cancer. Drugs.

[24] Bear HD, Anderson S, Brown A (2003). The effect on tumor response of adding sequential preoperative docetaxel to preoperative doxorubicin and cyclophosphamide: preliminary results from National Surgical Adjuvant Breast and Bowel Project Protocol B-27. J Clin Oncol.

[25] Heys SD, Hutcheon AW, Sarkar TK (2002). Neoadjuvant docetaxel in breast cancer: 3-year survival results from the Aberdeen trial. Clin Breast Cancer.

[26] Talbot DC, Moiseyenko V, Van Belle S (2002). Randomised, phase II trial comparing oral capecitabine (Xeloda) with paclitaxel in patients with metastatic/advanced breast cancer pretreated with anthracyclines. Br J Cancer.

[27] Greil R, Moik M, Reitsamer R (2009). Neoadjuvant bevacizumab, docetaxel and capecitabine combination therapy for HER2/neu-negative invasive breast cancer: efficacy and safety in a phase II pilot study. Eur J Surg Oncol.

[28] Jinno H, Sakata M, Hayashida T (2000). A phase II trial of capecitabine and docetaxel followed by 5-fluorouracil/epirubicin/cyclophosphamide (FEC) as preoperative treatment in women with stage II/III breast cancer. Ann Oncol.

[29] Madjar H, Ladner HA, Sauerbrei W, Oberstein A, Prompeler H, Pfleiderer A (1993). Preoperative staging of breast cancer by palpation, mammography and high-resolution ultrasound. Ultrasound Obstet Gynecol.

[30] Kald BA, Boiesen P, Ronnow K, Jonsson PE, Bisgaard T (2005). Preoperative assessment of small tumours in women with breast cancer. Scand J Surg.

[31] Lorendo V, Bazzocchi D, Del Frate C (2004). Locally advanced breast cancer: comparison of mammography, sonography and MR imaging in evaluation of residual disease in women receiving neoadjuvant chemotherapy. Eur Radiol.

[32] Huber S, Medl M, Vesely M, Czermbierk H, Zunna I, Delorme S (2000). Ultrasonographic tissue characterization in monitoring tumor response to neoadjuvant chemotherapy in locally advanced breast cancer. J Ultrasound Med.

[33] Bruno D, Fornage MD, Olivier T, Michel M (1987). Clinical, mammographic, and sonographic determination of preoperative breast cancer size. Cancer.

[34] Moneer M, El-Didi M, Khaled H (1998). Breast conservative surgery: is it appropriate for locally advanced breast cancer following downstaging by neoadjuvant chemotherapy?: A pathological assessment. Breast.

[35] Cocconi G, Dil Blasio B, Alberti G, Biagni G, Botti E, Peracchia G (1984). Problems in evaluating response of primary breast cancer to systemic therapy. Breast Cancer Res.

[36] Wenzel C, Bartsch R, Locker GJ (2005). Preoperative chemotherapy with epidoxorubicin, docetaxel and capecitabine plus pegfilgrastim in patients with primary breast cancer. Anticancer Drugs.

[37] Oshaughnessy JA, Blum J, Moiseyenko V (2001). Randomized, open-label, phase II trial of oral capecitabine (Xeloda) vs. a reference arm of intravenous CMF (cyclophosphamide, methotrexate and 5-fluorouracil) as first-line therapy for advanced/metastatic breast cancer. Ann Oncol.

[38] Cassidy J, Twelves C, Van Custen E (2003). First-line oral capecitabine therapy in metastatic colorectal cancer a favourable safety profile compared with intravenous 5-Fluorouracil/Leucovorin. Ann Oncol.

[39] Vancustem E, Findlay M, Osterwald B (2000). Capecitabine, an oral fluoropyrimidine carbamate with substantive activity in advanced colorectal cancer: results of a randomized phase II study. J Clin Oncol.

